# Electrospun Nanofibers: Shaping the Future of Controlled and Responsive Drug Delivery

**DOI:** 10.3390/ma16227062

**Published:** 2023-11-07

**Authors:** Michael Wildy, Ping Lu

**Affiliations:** Department of Chemistry and Biochemistry, Rowan University, Glassboro, NJ 08028, USA; wildym28@rowan.edu

**Keywords:** electrospun nanofibers, drug delivery systems, biopolymer membranes, stimuli-responsive materials, controlled release

## Abstract

Electrospun nanofibers for drug delivery systems (DDS) introduce a revolutionary means of administering pharmaceuticals, holding promise for both improved drug efficacy and reduced side effects. These biopolymer nanofiber membranes, distinguished by their high surface area-to-volume ratio, biocompatibility, and biodegradability, are ideally suited for pharmaceutical and biomedical applications. One of their standout attributes is the capability to offer the controlled release of the active pharmaceutical ingredient (API), allowing custom-tailored release profiles to address specific diseases and administration routes. Moreover, stimuli-responsive electrospun DDS can adapt to conditions at the drug target, enhancing the precision and selectivity of drug delivery. Such localized API delivery paves the way for superior therapeutic efficiency while diminishing the risk of side effects and systemic toxicity. Electrospun nanofibers can foster better patient compliance and enhanced clinical outcomes by amplifying the therapeutic efficiency of routinely prescribed medications. This review delves into the design principles and techniques central to achieving controlled API release using electrospun membranes. The advanced drug release mechanisms of electrospun DDS highlighted in this review illustrate their versatility and potential to improve the efficacy of medical treatments.

## 1. Introduction

### 1.1. Electrospun Drug Delivery Systems

**Figure 1:**
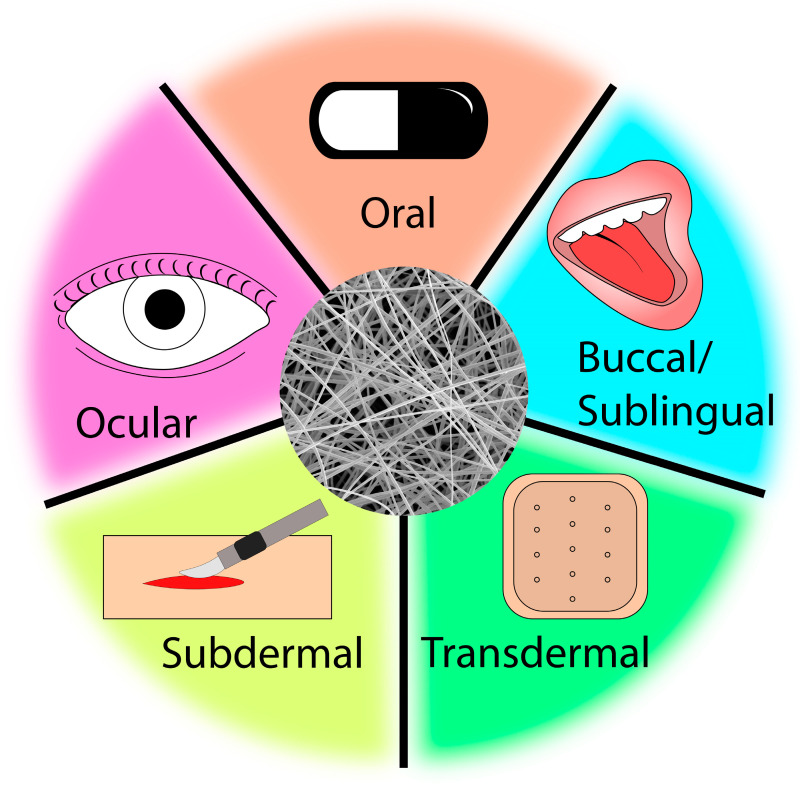
Representative modes of drug administration using ES DDS.

Electrospun (ES) drug delivery systems (DDS) have emerged as promising drug delivery vehicles suitable for a variety of modes of administration to treat a range of conditions (Figure 1) [1,2,3,4]. ES membranes are composed of randomly oriented fibers with diameters on the scale of micrometer to nanometer. Nanofibers possess unique properties, including a high surface-to-volume ratio, structure mimicking the extracellular matrix (ECM), and inter/intrafiber porosity [5,6,7]. Additional advantages of ES DDS are their high loading and encapsulation efficiency as well as scalability [8,9]. The nanofiber composition and property can be tuned using several methods to further adjust the release of drug payloads. One factor affecting the drug absorption rate is the administration route. The superior properties of nanofibers allow for unique drug release kinetics and biocompatibility of ES DDS. ES DDS afford increased versatility by offering novel modes of delivery for traditional medications. ES DDS for applications ranging from transdermal [10,11,12,13,14], subdermal [15,16,17], sublingual [18,19], buccal [19,20], vaginal [21], ocular [22,23], and oral [24,25] have been reported. Patients can benefit from improved delivery of pharmaceuticals to the active site and thereby enhancing therapeutical efficacy while simultaneously reducing systemic toxicity risks. The lure of improved patient outcomes and diminished side effects may lead to improved patient perceptions and increased interest or willingness to pursue treatment. Moreover, ES DDS also has profound commercial implications for the pharmaceutical industry as a means of improving profitability. The extension of revenue streams from drugs with expired patents allows for companies to increase market share without additional costs associated with the development of a new drug molecule.

### 1.2. Pharmacology and Controlled Release

The goal of controlled drug delivery is to transfer the correct amount of the active pharmaceutical ingredient (API) to the active site. This can be achieved by targeting specific windows for drug absorption to increase bioavailability, with most controlled-release drug delivery systems (DDS) developed to produce a sustained or pulsated drug release to specific parts of the gastrointestinal (GI) tract. The function of the GI tract is to absorb nutrients. Still, it is also an obstacle to overcome in drug delivery as degradation and metabolism of API results in its lower bioavailability. Gradual delivery to the upper GI tract is more important for drug bioavailability than direct gastric absorption in the stomach [26].

Most pharmaceutical formulations are prepared for oral drug delivery since it is non-invasive, cost-effective, convenient, and patient-friendly. However, some problems associated with oral medication are fluctuating plasma concentrations and non-specificity of drug delivery. The need for frequent administration of medication can result in a lack of patient compliance or contribute to overdose and systemic toxicity [26]. ES DDS for oral administration have been reported, such as water-soluble polymers for immediate release of drug [24] and insoluble polymers for sustained release of API [27]. The localized delivery of API is optimal for treating diseases that are concentrated in a particular organ or body area, such as cancer treatments and skin conditions. The flexibility and biocompatibility of ES membranes make them great candidates for transdermal applications as wound dressings and show promise for improving healing rates [13,14]. Transdermal delivery is classified as parental as it does not involve drug absorption through the GI tract and allows for localized drug delivery. Implantable DDS are marketed for the continuous release of API over long periods. Systemic toxicity is especially an issue with chemotherapy drugs since they traditionally exploit the rapid growth of cancer cells, but the selectivity is insufficient to completely avoid killing healthy cells [28,29]. The severe side effects of chemotherapy drugs warrant a cost-benefit analysis before starting a treatment regimen [30]. A more efficient delivery of therapeutic agents to the active site would be beneficial to reducing the unwanted side effects. ES membranes have been gaining attention as potential implantable scaffolds for the extended release of chemotherapy drugs at the site of action post-surgical tumor removal [15,16,17].

The most efficient mode of delivery of pharmaceuticals is typically determined by the nature of the disease and the drug properties and bioavailability. Bioavailability can be vastly increased by improved controlled release of drugs [26]. While oral drug formulations dominate the market, their efficiency in facilitating the absorption of various types of drugs leaves room for improvement. For example, stabilization of amorphous forms of API can augment drug absorption rate and extend shelf life [31]. Electrospun membranes represent a viable alternative for drug delivery vehicles with the potential for enhanced pharmacokinetics. Variables in the fabrication of electrospun membranes such as polymer selection, solvent, and inclusion of stimuli-responsive materials as well as nanocarriers enable fine-tuning of drug release rate that can achieve improved targeted drug delivery and drug efficacy. The numerous possibilities for modification and adjusting of drug release kinetics offers the potential for highly selective and/or localized drug delivery.

### 1.3. Electrospinning

**Figure 2:**
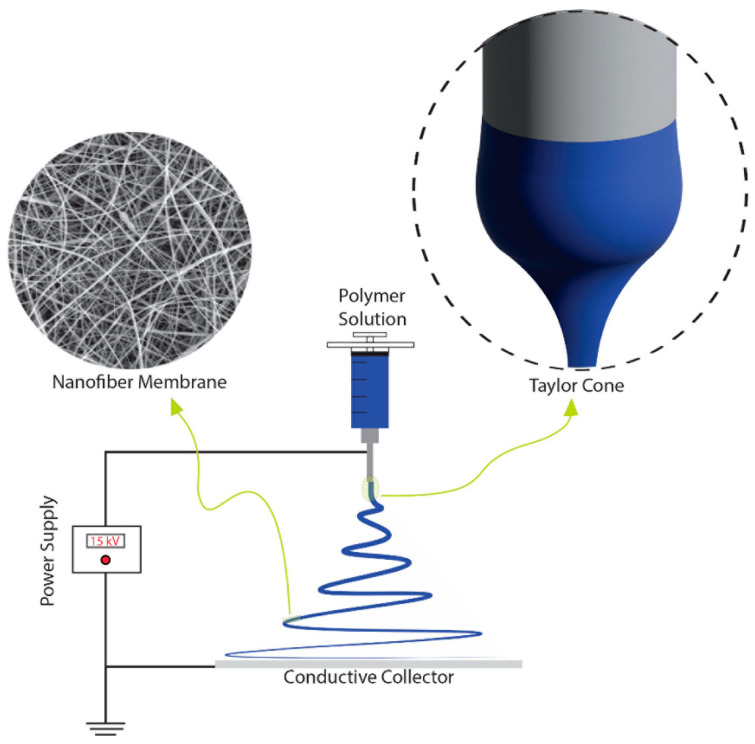
Schematic illustrating the uniaxial electrospinning apparatus setup. Image was drawn on Adobe Illustrator.

Electrospinning is a straightforward method for fabricating nonwoven or aligned continuous polymer fibers. A schematic diagram of a uniaxial electrospinning setup in a vertical orientation (Figure 2) is shown below. The process uses a high-voltage power supply to stretch fibers as a polymer solution in a syringe is forced through a needle. The DC power supply can be adjusted from roughly 1–30 kV, and a syringe pump controls the solution feed rate. The high-voltage power supply is connected to the metal needle, which causes a buildup of Coulombic forces in the charged polymer solution and forces the spinneret droplet to elongate into a Taylor cone. The repulsive forces further cause a polymer jet to eject and accelerate from the tip of the Taylor cone. Elongation and coiling of the polymer jet are caused by instability and pulling towards the conductive collector due to the repulsive Coulombic forces. Rapid solvent evaporation and polymer solidification produce the membrane, which is deposited on the conductive metal collector positioned at a set distance from the needle tip [32]. 

Uniaxial electrospinning uses a single polymer solution and is the most straightforward configuration. Coaxial electrospinning involves the use of multiple polymer solutions to form fibers with core-sheath composite structures. Electrospinnable polymers include polystyrene (PS) [33], poly(methyl methacrylate) (PMMA) [34], poly(vinyl alcohol) (PVA) [2], polylactic acid (PLA) [35], polyvinylpyrrolidone (PVP) [25], polycaprolactone (PCL) [36], among others. Environmental and processing factors, such as relative humidity, temperature, polymer concentration, solvent, solution feed rate, applied voltage, needle tip diameter, tip-collector distance, etc., can influence the properties of electrospun nanofibers [32]. Electrospun membranes have shown promise in a variety of applications, including catalysis, sensors, filtration, energy storage, tissue engineering, and drug delivery, as well as others [15,37,38,39,40,41]. One practical consideration from a commercial aspect is the scalability of electrospinning. A single typical lab scale electrospinning setup is only capable of yielding roughly 5–15 g per day, depending on feed rate and polymer concentration, while kilogram scale is required during the production scale up phase of pharmaceutical manufacturing. One approach to increasing the production rate is the use of a spinneret with multiple needle tips being fed by a single polymer solution. Accordingly, this technique would be much easier to adopt for uniaxial electrospinning and interactions between the electric fields of the nozzles can affect fiber quality. Another method for increasing the production rate is the use of free surface methods instead of spinnerets with nozzle needles. This method allows for the generation of more Taylor cones and the potential for much higher yield. However, the use of volatile solvents in polymer solutions creates a changing concentration over time as the exposed solvent evaporates [42]. A spinneret with multiple orifices has been shown to eliminate the problems associated with exposed solvents. This type of free surface spinneret was reported to be capable of producing ~5 kg per day of drug-loaded fibers [43]. While most ES uses DC power supply, AC has been shown to provide some advantages for production scale-up. Multiple jets can be formed from a single droplet with an AC power supply, increasing productivity [42]. 

### 1.4. Biopolymers

Biopolymers are polymers that are naturally occurring or synthesized from precursors found in nature, and, thus, renewable resources. In addition, these abundant biopolymers have properties beneficial to pharmaceutical/biomedical applications, such as biocompatibility, biodegradability, and antibacterial properties [44]. The electrospinning of numerous types of biopolymers has been reported [45]. A major class of electrospinnable biopolymers are polysaccharides. Natural polysaccharides mainly include cellulose and derivatives, chitin, chitosan, and dextran. Another notable biopolymer class is proteins such as silk, collagen, and gelatin.

**Figure 3:**
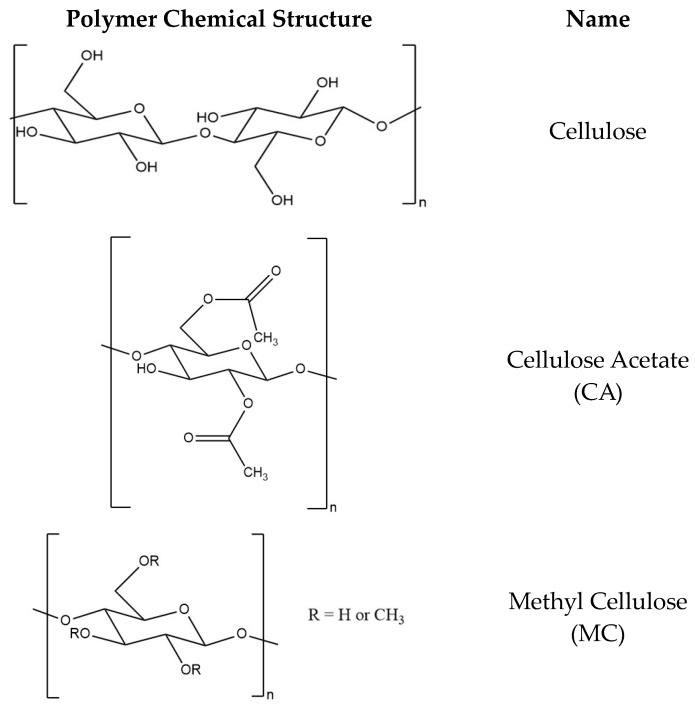

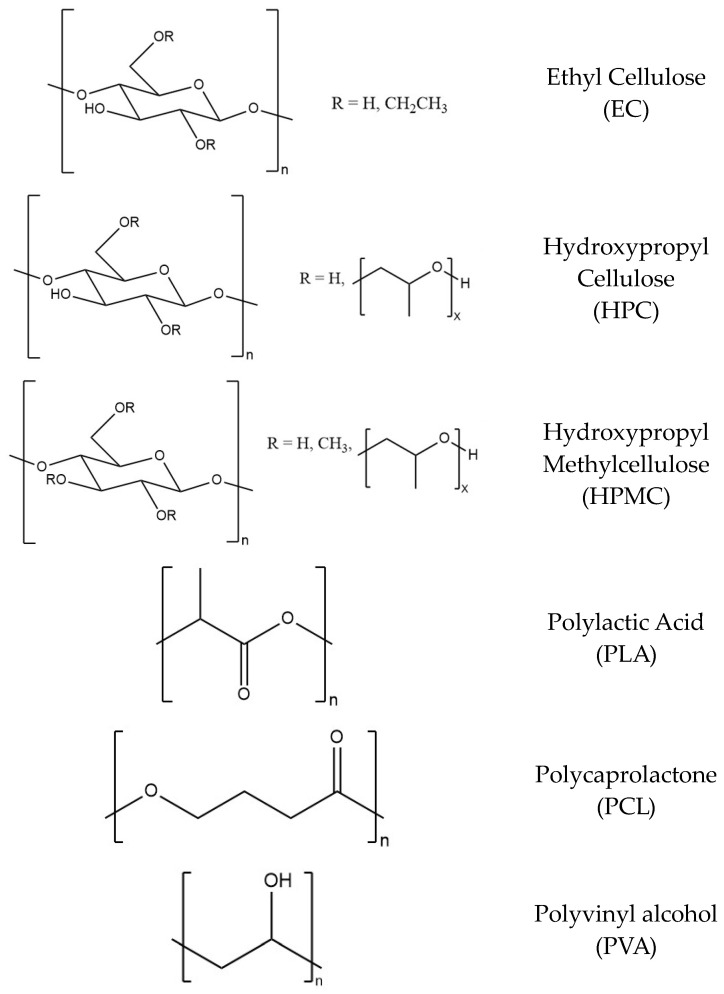
Chemical structures of cellulose, cellulose derivatives and other polymers commonly used in ES DDS [50,52,53].

Cellulose is the most plentiful natural resource and the main biopolymer component in lignocellulosic biomass, the most abundant type of biomass, with an estimated 181.5 billion tons produced annually [46]. Cellulose is very widely available since it is the main component of plant cell walls. The chemical structure of cellulose (Figure 3) contains a linear polysaccharide containing repeating glucose units, which are connected by linkages at the β-(1→4) positions. Each glucose unit has three hydroxyl groups, which can be reacted via substitution reactions to synthesize various cellulose derivatives. The insolubility of cellulose in most organic and inorganic solvents precludes the use of cellulose as a polymer for electrospinning. The material properties of cellulose can be improved by derivatization. Some common cellulose derivatives that can be electrospun include cellulose acetate (CA) [47], methylcellulose (MC) [48], hydroxypropyl cellulose (HPC) [49], hydroxypropyl methylcellulose (HPMC) [20], and ethyl cellulose (EC) [50]. Vueba et al. studied the effect of excipients by comparing dissolutions of tablets with formulations of ketoprofen (KET) with MC, HPC, and HPMC. The release mechanism of KET was found to be independent of these polymers, and the highest mean dissolution time (MDT) was obtained from HPMC polymer formulation tablets (zero-order) [51].

## 2. Controlled Release Electrospun Drug Delivery Systems

### 2.1. Electrospun Nanofibers for Drug Delivery

**Figure 4:**
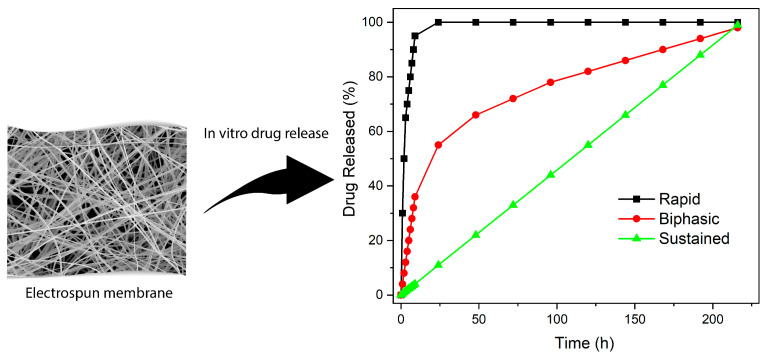
Illustrative release profiles generated for comparative analysis. This figure, created with OriginPro, serves as a visual guide to typical drug release behaviors. The patterns depicted are not derived directly from experimental data; hence, both axes are marked with arbitrary units to conceptualize the various release types.

There are several techniques employed with ES DDS to achieve controlled API delivery. The properties of the polymer can be altered by blending multiple polymers in a single solution, using multiple polymer solutions, or using various solvent systems. Another method to control the release of API is using additional materials capable of bonding to the drug or encapsulation [54,55], such as nanocarriers [56,57]. The choice of polymers and the processing parameters play a significant role in the release mechanism. The polymer properties contributing to the release of API are swelling in water, polymer-drug affinity, and degradation rate. Several studies have reported the effect of polymer type on drug release kinetics. Researchers compared the release of ciprofloxacin HCl (CIF), a fluoroquinolone antibiotic, from polyvinyl acetate (PVAc) and polyvinyl alcohol (PVA) [2]. The hydrophilic property of PVA produced a vastly different release profile, with a complete release in less than an hour for PVA compared to up to a week for PVAc. The study illustrated the pivotal role that polymer choice plays in drug release kinetics. A comparison of typical rapid, biphasic, and sustained release profiles is shown in Figure 4.

One of the most straightforward drug delivery applications using ES nanofibers is enhancing the dissolution rate of poorly soluble APIs. Some examples include incorporating ibuprofen (IBU) [58] and KET [59] in hydrophilic polymers such as PVP [24], PVA [2], and CA [3]. This was achieved by fabricating nanofibers containing amorphous solid dispersion (ASD) of API instead of the more energetically favorable crystal structure of the bulk API material [60]. The lack of crystal lattice lowered the energy barrier for the dissolution of the poorly soluble API into the release medium. Accordingly, the release rate was vastly improved compared to the bulk API in pure crystalline form [10,24,25,31]. The enhanced dissolution of API can improve the bioavailability of poorly soluble drugs, which can provide immediate relief and achieve maximal efficacy with lower dosages. Lopez et al. reported PVP fibers loaded with amorphous distributions of indomethacin (IMC) and griseofulvin (GSF) that showed significant immediate release of API and excellent stability with API retaining amorphous form after eight months [31]. The authors reported an amorphous distribution of API when loaded up to 33.33% w/w after optimizing the process parameters for electrospinning. The drug degree of loading while maintaining amorphous state of API demonstrates the profound stabilization effect possible using ES DDS and the correct choice of polymer and drug. Andriotis et al. reported the first ES DDS for the rapid delivery of cannabinoids cannabidiol (CBD) and cannabigerol (CBG) using PVP and Eudragit polymers [41]. Their work demonstrated a potential immediate release of DDS for the poorly soluble cannabinoids to be administered orally. 

Sustained release of API can be achieved by controlling the matrix hydration rate and drug diffusion rate from the polymer matrix. Hydrophobic polymers are excellent candidates for delayed and sustained release of DDS owing to their low wettability [2]. Some hydrophobic polymers used in ES DDS designed for sustained release include EC [27,61], PLA [62], poly-lactic-co-glycolic acid (PLGA) [63,64,65], polyurethane (PU) [66], and PCL [67]. Sustained drug release kinetics can be advantageous for achieving efficient drug delivery using a variety of modes of administration. Factors to be considered for designing sustained-release DDS include polymer type, drug and its physicochemical property, and formulation property. Wu et al. studied the release kinetics of CIF from PLGA [63]. The authors proposed three phases of drug release kinetics. The first phase, lasting only a few hours, followed first-order release kinetics and was controlled by polymer swelling. The second phase followed zero-order release kinetics and was controlled by drug diffusion to the fiber surface, lasting several days. The enzymatic degradation of PLGA facilitated the final stage of CIF release. Polymer-drug interactions in both solution and solid form affect the dispersion of API and release rate. Therefore, the physicochemical properties of drugs are essential to consider since small differences can lead to disparities between the release of various drugs. Accordingly, the insight gained from release profiles of model drugs from a DDS may offer limited relevance concerning the use of other drugs [68]. The properties and interactions between the drug, polymer, and release medium all contribute to the release kinetics; therefore, ES DDS should be studied with the intended target drug to ensure consistent results.

EC is an ethyl ether cellulose derivative synthesized by substation of 44–51% of cellulose’s hydroxyl groups. The viscosities of EC solutions depend on the polymeric backbone chain length, with viscosity increasing with chain length. EC polymer can withstand the release of drugs and is included in tablet formulations as a binding agent and thickener. This thermoplastic cellulose-derived polymer is biocompatible, biodegradable, light and heat-resistant, nontoxic, colorless, and tasteless. EC is frequently used in the pharmaceutical, cosmetic, and food industries [69]. The water-insoluble polymer is commonly used for coatings of solid-dosage form and masking taste. EC is employed in modified-release dosage form due to its hydrophobic properties and low swelling capacity. Pharmaceutical effectiveness can be improved by controlled release of drugs at a steady rate to maintain consistent drug blood concentrations. EC coatings allow for extended, modified release of pharmaceuticals due to the hydrophobic nature limiting the wetting of the polymer matrix. The release rate of drugs can also be adjusted with the use of EC polymers with different molecular weights [70]. The encapsulation of API in EC microspheres [71], microcapsules [72], and films [69,73] for controlled release applications has been reported. EC nanofibers have the potential for modified release and targeted drug delivery due to the insoluble and biodegradable nature of the polymer. 

EC is commonly used as a polymer in controlled-release ES DDS. EC’s hydrophobic and insoluble properties limit the API diffusion rate from the polymer matrix [12,27,74,75,76,77,78,79,80,81,82,83,84]. The addition of varying ratios of EC to composite ES DDS can tune the release kinetics of API for various drug delivery applications. Composite nanofibers DDS consisting of EC and hydrophilic polymers such as PVP [12,20] and polyethylene oxide (PEO) [81,85], as well as zein [86], have been reported. Lipophilic drugs, such as KET [80,87], IBU [85], and IMC [20], have good compatibility with EC, stabilizing the API in amorphous form. A benefit of using additional polymers with EC is the improved electrospinnability over EC alone. Additionally, many examples of coaxial ES of EC and other polymers, most commonly PVP, have been reported [75,76,78,79]. EC polymer is widely used as the sheath to create sustained-release DDS. Ball et al. reported coaxially ES PVP core and EC sheath fibers loaded with antiviral maraviroc. The release of API was shown to be biphasic and adjustable by varying the thickness of the EC outer layer [79]. The sustained release of quercetin over eight days from zein core-EC sheath fibers has been reported [83]. Nouri et al. reported the simultaneous release of two chemotherapy agents from 2-D nanosheets encapsulated in chitosan core-EC sheath fibers [88]. The DDS showed continuous zero-order release of API over 20 days. Another reported ES DDS produced from EC used bead-on-string morphology to achieve controlled release [76].

ES solutions containing a blend of polymers, typically hydrophilic/hydrophobic, in varying ratios, are used to control the release rate of the drug to enhance therapeutic efficiency [12,34,74,89]. By changing the ratio of different polymers/materials, the properties of the composite fibers can be tuned to control the release of API [74,85,89]. The release can be fine-tuned to better suit the application of the specific drug being delivered. An initial burst release is undesirable in most drug delivery applications and can be reduced by adding hydrophobic polymers to achieve biphasic or sustained release of API. EC is commonly blended with other polymers to reduce the initial burst release and improve the sustained release of API [14,20,74,80,85]. However, in cases such as treating a bacterial infection, a burst release is beneficial for patient outcomes [90]. A biphasic release consisting of an initial burst followed by a sustained, steady release of the loaded drug can be obtained by finding an ideal polymer ratio [34,80,89]. A blend of CA and EC polymers was reported to be used to produce ES DDS with tunable burst and sustained release rates [80]. Alternatively, the release rate can be increased by incorporating a water-soluble polymer, such as gelatin [36]. Table 1 summarizes DDS fabricated by uniaxial ES from blends of polymers, drugs, and nanocarriers/surfactants for various controlled drug release applications.

The morphology of the ES DDS can affect the interaction between the polymer matrix and the release medium. Fibers with bead-on-string structures have generally been regarded as low quality; however, they may prove useful in drug delivery applications. The morphology of bead-on-string fibers creates varying polymer thickness throughout the mat and, thus, simultaneous dual passive diffusion rates of API from the polymeric matrix [76,92]. Porosity can also be used to control the diffusion rate of API from the DDS. The porosity of ES fiber can be controlled using various phase separation methods. Solvent choice plays the most significant role in forming pores in the interior and on the surface of ES fibers. Adding a nonsolvent can cause the de-mixing of polymer in the solution and the formation of pores [93]. ES of PU in DMF was reported to produce porous fibers that demonstrated a biphasic release of itraconazole [11]. Binary solvent systems composed of solvents with contrasting properties can be used to tune porosity and control the release of API from the ES DDS [94,95]. This can increase the surface area and improve the diffusion of API from the hydrophobic polymer matrix by allowing greater penetration of the release medium [96,97]. 

The method used for the loading of API into ES DDS can determine drug distribution and affect drug release kinetics. The most straightforward drug loading method is mixing in a blend ES polymer solution. Another way is emulsion ES by adding surfactants to form micelles in the polymer solution. Micelles have been studied for their usefulness as drug carriers, which are compatible with hydrophilic and hydrophobic model drugs [98,99]. Emulsion ES can encapsulate drug-containing micelles in the polymer for controlled release [64,100]. Drugs can also be loaded into ES fibers after fabrication via absorption of API in solution [101].

**Figure 5:**
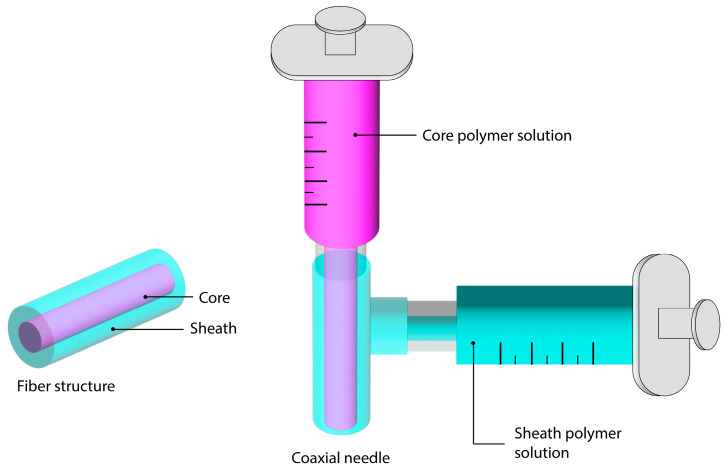
Diagram of coaxial electrospinning and core-sheath fiber structure (drawn on Adobe Illustrator).

Another method is core loading in multi-layered fibers. Composite ES fibers made of a mixture of polymers can also be fabricated by feeding multiple solutions to a coaxial ES spinneret, producing core-sheath fibers, as shown in Figure 5. The ratio of polymers can be easily adjusted in coaxial needles by varying the solution feed rate ratios, resulting in varying core and sheath layer thickness. Hydrophobic polymers, such as EC, as the sheath polymer, and hydrophilic polymers, such as PVP or other polymers, as the core polymer, have been reported [79,83]. Core loading of API also enables control of burst release with the additional outer layer barrier and distance for the diffusion of API. The coaxial method of fabricating ES DDS with blends of multiple polymers has been shown to biphasic [75,79], sustained [102,103], rapid [104], or stimuli-responsive [102,105] release of API. Coaxial ES has been reported to be used to produce fibers that demonstrated the controlled release of two model drugs by loading each in separate layers [106]. The use of coaxial ES also allows for opportunities in fine-tuning other properties, such as taste, by controlling the core and sheath thickness. The optimization of taste-masking properties using two polymers for rapid release from oral films using coaxial ES has been reported [107]. Modified triaxial ES using three solutions, a core of KET, an inner of EC and KET, and an outer of ethanol, showed a reduction in burst release compared to a blend of EC and KET [108]. Coaxial and triaxial ES DDS are increasingly complex compared to uniaxial and it is imperative that the electrospinning process is sufficiently optimized to produce good quality and consistent fibers. The interaction between multiple solutions can greatly affect the ease of ES. The interfacial tension between solutions should be minimized, which can be easily achieved with miscible core and sheath solutions [109]. Recent coaxial ES fibers for controlled release in drug delivery applications are summarized in Table 2.

### 2.2. Chemotherapy Drugs

Doxorubicin HCl (DOX) is an anthracycline common chemotherapy drug administered by IV to patients with various forms of cancer, including sarcomas, carcinomas, and hematological [112]. DOX exhibits several mechanisms of action. One mechanism is the inhibition of macromolecular biosynthesis by interacting with DNA through intercalation, preventing DNA replication [113]. Another mechanism of action of DOX is the inhibition of topoisomerase II by stabilizing enzyme-DNA intermediates during replication [91]. DOX can also form free radicals capable of damaging cell membranes and DNA. There is a need for improved selective delivery of DOX to the desired site to increase its efficiency and patient outcome. Serious side effects can lead to diminished quality of life as well as death for patients. This is due to injury of healthy tissues and vital organs, such as the heart, liver, kidney, and brain [112]. An improved delivery system for DOX could alleviate unwanted side effects and increase the drug’s efficacy. Localized administration would be beneficial to avoiding systemic toxicity associated with intravenous drugs. This method of treating tumors offers additional localized drug delivery potential due to the metabolic microenvironment in tumors. Some unique properties, such as acidosis and hypoxia, could be exploited as triggers for the targeted delivery of chemotherapy drugs [114]. Therefore, stimuli-sensitive ES DDS presents an emerging technique for localized chemotherapy delivery targeting tumor sites. 

Several reported ES DDS containing DOX have been reported for the sustained release of DOX [110,111,115]. The release of DOX from PLA ES DDS has been widely reported with or without nanocarriers, such as graphene oxide and mesoporous silica nanoparticles (MSNs) [17,56]. PLA or PLA polymer blends have been used to fabricate ES DDS containing DOX as implantable scaffolds [15,16,116]. Gohary et al. reported a coaxial ES DDS using a core solution containing PEO, DOX, and PCL sheath. The release rate was shown to be controlled by varying the polymer solution feed ratios. A lower burst release over blend ES and increased in vitro efficacy were observed compared to pure DOX [110]. However, a biphasic release of chemotherapy drugs may be more efficient in suppressing tumor growth. An ES DDS made of a PEO and PLA polymer blend showed biphasic DOX release and good in vivo cancer treatment efficacy [15]. The treatment of breast cancer tumor recurrence by release of DOX from MSNs embedded in PLA nanofibers has been reported. In vitro drug release experiments showed a biphasic release and in vivo studies demonstrated tumor inhibition and sustained apoptosis of tumor cells over 10 weeks [117]. The dual release of angiogenesis inhibitor apatinib (Apa) and DOX from triaxial ES PLA/PCL fibers with the application of implantable scaffold for breast cancer treatment has been investigated. DOX was concentrated in the center, encapsulated by a PLA middle later and further wrapped by a PCL sheath layer containing Apa. An in vivo cytotoxicity assay revealed good efficacy and tumor blood vessel inhibitory effect as well as low systemic toxicity [118]. The efficacy of paclitaxel on the inhibition of tumor growth was shown for the ES DDS composed of poly(glycolide-ԑ-caprolaction) (PGCL)/PLGA blend fibers. Monitoring of tumor size during in vivo studies indicated a significant volume reduction and good efficacy of the drug loaded fibers [119]. This study demonstrates the utility of implantable ES scaffolds for inhibition of tumor growth without prior tumor removal surgery. 

Another common chemotherapy agent is 5-Fluorouracil (5-FU). 5-FU is classified as a antimetabolic with a primary mechanism of action of inhibition of thymidylate synthase resulting in the interruption of nucleotide synthesis [120]. The release and cytotoxic effect of 5-FU from coaxial ES DDS composed of 5-FU-loaded PVP core and PLGA sheath was reported [121]. A tunable drug release and good anti-tumor effects were observed. The drug can also be administered in combination with other chemotherapy drugs to improve patient outcomes by achieving a synergetic effect. The dual release of 5-FU and methotrexate (MTX) was reported from PCL/chitosan blend ES DDS with control of drug release rate achieved by concentrating the majority of drugs in the inner most layer surrounded by layers of fibers with various drug loading [122]. The delivery of 5-FU from an PLA/gelatin ES DDS in the form of a patch was combined with the systemic administration of gemcitabine, another chemotherapy drug, to study their synergistic effect [123]. The researchers compared three groups: a control of patch without any drug, 5-FU-loaded patch, and a combination of drug-loaded patch and systemic administration of gemcitabine. The anti-tumor effects were studied using in vitro and in vivo models. The use of a 5-FU-loaded patch and combined with administration of gemcitabine demonstrated an improvement of therapeutic effects over the use of only the 5-FU-loaded patch. These studies show the usefulness of combining different drugs, through either the same route of administration or different routes, in enhancing the therapeutic effect.

### 2.3. Biocompatibility

An intrinsic property of biopolymers and biomaterials is their biocompatibility. The nano- to micrometer scale of ES fibers also allows for unique properties. One such property is the physical structure of ES membranes created by the overlaying of randomly oriented fibers, which mimics the ECM, allowing for enhanced biocompatibility and facilitation of cell proliferation [124]. Numerous in vitro cytotoxicity/cell viability assay studies have verified the nontoxic nature of biopolymer-based ES DDS [13,14,17,65,102]. A cell proliferation study using in vitro MTT assays showed good biocompatibility and a correlation between fiber size and cell adhesion and proliferation [125]. Membranes containing fibers with smaller diameters were found to allow for greater interaction with cells due to larger surface area and can enhance cell proliferation. Furthermore, the addition of biopolymers to blend ES DDS has been shown to increase the biocompatibility and cell proliferation rate [125]. Excellent attachment of MCF-7 and HeLa cells to drug-loaded chitosan-EC core-sheath fiber membranes has also been shown [88]. 

## 3. Advanced Controlled Release Drug Delivery Systems

Additional materials have also been incorporated into ES DDS to enhance the selectivity of drug delivery and achieve greater therapeutic effect. Nanocarriers have gained attention for their high drug encapsulation efficiency and biocompatibility [10]. Some examples of nanocarriers include metal-organic frameworks (MOF) [54] and 2-D materials like graphene oxide (GO) [57] and MXene [126]. Interactions like π−π stacking (hydrophobic interaction) and hydrogen bonding can bond the drug to a nanocarrier, such as GO [57]. Some nanocarriers are sensitive to external stimuli, such as pH and temperature, allowing for a trigger for the release of API. The stability of the MOF can be readily adjusted using different metals. Zeolitic imidazolate framework (ZIF-8) [54,127] is a MOF that has gained attention for its usefulness in targeted drug delivery owing to its nontoxicity and instability at low pH conditions. Magnetism can also be used for targeted release of API from magnetic nanoparticles [128] and composite nanofibers [129]. 

**Figure 6:**
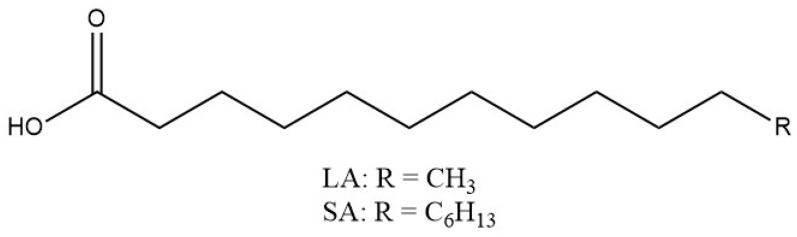
Lauric acid and stearic acid chemical structures.

Phase change materials (PCMs) are commonly used for thermal energy storage (TES) systems with the application of storing latent energy [130]. The latent heat of fusion is absorbed or released by PCMs during the freezing or melting processes [131]. PCMs can be selected with phase transition temperature ranges suitable for the desired applications. Recently, researchers have aimed at utilizing PCMs to create composite nanofibers possessing melting points slightly higher than the physiological body temperature to allow for the controlled release of various types of drugs. Fatty acids (FA) are a class of PCMs suitable for DDS due to their biocompatibility, biodegradability, and stability. FA eutectics, mixtures of two or more fatty acids in specific ratios, have lower melting points than FA alone. The chemical structures of saturated fatty acids lauric acid (LA) and stearic acid (SA) contain 12 and 18 carbon chains, respectively (Figure 6). The aliphatic carbon chains of LA and SA allow for hydrophobic nature that can aid in minimizing drug release when the PCM is solid. The LA/SA eutectic mixture consisting of a 4:1 ratio has been reported to possess a sharp melting point around 39 °C [132,133] slightly above the normal human physiological temperature of 37 °C. LA/SA in composite material DDS can be melted at a temperature slightly above the eutectic FA mixture m.p. (i.e., 40 °C) to allow diffusion of API through the PCM and achieve targeted release of drug payload [55]. 

Various types of DDS containing PCM have been reported. Earlier, PCM-DDS used hydrogels to encapsulate PCM and drugs [134]. Thermally sensitive polymers, such as poly(N-isopropyl acrylamide), have been used to produce hydrogels as stimuli-sensitive DDS and incorporated into blend ES DDS [77,135]. However, one drawback of hydrogels is their lack of drug release control. Micro- and nanoparticle DDS containing PCM have also been reported [55,136]. Gelatin particles containing fluorescein isothiocyanate-dextran (FITC-dextran) were produced by emulsion and encapsulated in microbeads of 1-tetradecanol, a PCM with a melting point of 38–39 °C. Drug release from the microbeads was shown to be negligible at 37 °C over 24 h, yet quickly increased to nearly 100% after increasing the temperature to 39 °C. The effect of temperature response on drug delivery was also shown to depend on the polymer, with the highest release rate being gelatin microbeads, followed by chitosan and PLGA, respectively [136]. Vitamin E was reported to be encapsulated in a eutectic mixture of LA/SA (3:1) nanocapsules with a particle size distribution (PSD) of 70–550 nm. The immediate release of vitamin E upon heating to 37 °C was confirmed visually [137]. Pan et al. reported using ES DDS consisting of a blend of Eudragit^®^ RS 100 and poly(methyl methacrylate) for the temperature-responsive delivery of antimicrobial octenidine. However, the thermal switch “on” temperature of 37 °C, while the “off” temperature was 25 °C, was observed [138]. Hu et al. reported the incorporation of thermally sensitive polymer poly(N-isopropyl acrylamide) (PNIPAAm) in a blend with EC [77]. The ES DDS’ properties displayed a switch from hydrophilic to hydrophobic when the temperature was increased above the critical temperature of 32 °C.

The use of a eutectic mixture of LA/SA (4:1) has been reported in several ES DDS. Inorganic nanomaterials, including hollow SiO_2_ nanoparticles with holes in the wall [139], mesoporous SiO_2_ [140], and gold nanocages [141], have been reported for the encapsulation of PCM for stimuli-sensitive DDS. Qiu et al. reported the temperature-responsive delivery of doxorubicin using LA/SA (4:1) encapsulated in silica-based nanocapsules with well-defined hole-in-the-wall morphology [139]. Additionally, alternative modes of heating can also be used to control the release of API from DDS containing PCM. The combination of PCM and magnetic particles has been shown to control the release of API. Thermal energy is created when a magnetic field is applied, thus generating sufficient heat to melt the PCM and increase the release rate. Heat generated from magnetic nanoparticles has been reported [142,143]. High intensity focused ultrasound can also be used as a method of providing heat and control the release of API from PCM-DDS [144]. PCM can also be used to encapsulate API for stimuli-sensitive DDS. The temperature-responsive delivery of nearly 100% Rhodamine B (RhB) in 10 min at 40 °C from electrospray microparticles composed of LA/SA shell and gelatin and RhB core was reported [55]. Another PCM-DDS is nanofibrous hydrogels. Zhang et al. reported a temperature-responsive release of aspirin from nanofibrous hydrogels containing LA/SA in a 4:1 ratio [145]. Drug release after 48 h was found to be 44%, 56%, and 87% at 25 °C, 37 °C, and 40 °C, respectively. However, the sample preparation involved several post-ES steps. Gelatin methacryloyl (GelMA) nanofibrous hydrogels were electrospun and crosslinked under 365-nm ultraviolet lighting. LA/SA/aspirin was co-encapsulated in polydopamine (PDA) microspheres by oil–water emulsion, followed by Michael addition reaction between amino groups on GelMA and PDA at pH 8.5 to attach the microspheres to the nanofibrous hydrogels. 

## 4. Conclusions

Electrospinning stands at the forefront of advanced DDS fabrication, offering precise control over the release of API. Its environmentally conscious nature is underscored by its use of renewable biopolymers and benign solvents. The versatility of electrospun DDS opens new avenues for pharmaceutical administration, with the added benefit of stimuli-responsive systems that can adapt to drug target conditions, bolstering selectivity and reducing toxicity risks. The inclusion of electrospinning in API formulations holds the potential to elevate therapeutic efficacy across various diseases. As a consistent and scalable method, electrospinning is poised to revolutionize controlled-release formulations. The multifaceted applications and innovative release mechanisms of electrospun DDS herald their promising role in the pharmaceutical industry. Further clinical testing will allow for commercialization and awareness of the full potential that ES DDS offer.

## Figures and Tables

**Table 1:** Uniaxial electrospun DDS fabricated from blends of polymers, drugs, and additional materials.

Polymer/Materials	Drug	Release Profile	Ref.
EC	IMC	Sustained	[86]
EC	Quercetin	Sustained	[61]
EC/CA	IMC	Sustained	[80]
EC/PVP	Naproxen (Nap)	Sustained/tunable	[14]
EC, PVP	CIF	Sustained	[84]
EC/HPMC/Tween 80	IMC	Rapid	[20]
CA	Thymol (THY)	Sustained	[13]
PLA	DOX	Sustained	[16]
PLA/MSN	DOX	Sustained	[17]
PLA/GO	RhB	Controlled	[56]
PLGA	CIF	Biphasic	[63]
PLA/PLGA/Si nanoparticles	Gentamicin sulfate (GS)	Controlled	[35]
PLA/PEO	DOX	Biphasic	[15]
PCL	IBU	Sustained	[67]
PCL	Carvedilol (CVD)	Controlled/tunable	[18]
PCL/PEO/Si nanoparticles	DOX	Sustained	[91]
PVP	Cyclosporine A (CA)	Rapid	[25]
PVP	Metronidazole (MET)	Rapid	[21]
PVP/Eudragit RS100	CBD, CBG	Rapid	[41]
PVA, PVAc	CIF	Rapid	[2]

**Table 2:** Coaxial electrospun controlled release DDS consisting of various core-sheath polymer combinations and/or nanocarriers.

Core	Sheath	Drug	Release Profile	Ref.
PVP	EC	Maraviroc	Controlled/tunable	[79]
EC	PVP	Quercetin	Biphasic	[75]
Zein	EC	Quercetin	Sustained	[83]
EC	Ethanol	KET	Sustained	[87]
Chitosan	EC/nanosheets	DOX/FA	Sustained	[88]
PCL, PVA	PVP	Quercetin, Tamoxifen citrate (TC)	Rapid	[104]
PEO	PCL	DOX	Sustained	[110]
Carboxymethyl chitosan (CMC)	PCL	DOX	Controlled/Sustained	[102]
PLGA/PCL	Gelaton/Genipin	DOX	Controlled/Sustained	[103]
DOX	PLCL/Gelatin	DOX	Sustained	[111]
Polyethylene Gylcol (PEG)	PLA	Salicylic acid (SA)	Sustained	[96]
PEG	PU			
PVA	PMMA	CIP	Sustained	[4]
PVA	PCL	DOX	Controlled	[105]
Kollicoat^®^ Smartseal	Eudragit^®^ EPO	Chloropheniramine maleate	Rapid	[107]

## Data Availability

Data available on request from the authors.
